# Process-based Management aimed at improving health care and financial results

**DOI:** 10.1590/1980-220X-REEUSP-2021-0333en

**Published:** 2022-05-06

**Authors:** Fernanda Novaes Moreno Brancalion, Antônio Fernandes Costa Lima

**Affiliations:** 1 Universidade de São Paulo, Escola de Enfermagem, Programa de Pós-graduação em Gerenciamento em Enfermagem, São Paulo, SP, Brazil.; 2 Universidade de São Paulo, Escola de Enfermagem, Departamento de Orientação Profissional, São Paulo, SP, Brazil.

**Keywords:** Health Management, Total Quality Management, Value-Based Purchasing, Hospital Administration, Health Care Costs, Gestión en Salud, Gestión de la Calidad Total, Compra Basada en Calidad, Administración Hospitalaria, Costos de la Atención en Salud, Gestão em Saúde, Gestão da Qualidade Total, Aquisição Baseada em Valor, Administração Hospitalar, Custos de Cuidados de Saúde

## Abstract

The limited resources allocated to the health area and the growing demands require leaders’ qualified and committed performance in hospital management. In this perspective, the objective of this study is to reflect on the management practices that can be applied to hospital facilities to achieve better care and financial results. Among them, process-based management proposes an approach for continuous process improvement to achieve desired results; the method *Lean Six Sigma* allows identifying and eliminating waste in production processes; the continuous improvement model combines practical knowledge with the knowledge of how the system to be improved works, through observations and changes that allow its results measurement; and cost management and value-based healthcare provides for care mapping, from beginning to end, to assess what actually adds value to patients. The contributions of implementing these practices are recognized worldwide; using them, processes can be increased, improving efficiency, reducing waste, adding value to the business, increasing its revenue, and resulting in savings that can be passed on to the consumer, by improving quality.

## RESUMO

Os recursos limitados destinados à área da Saúde e as demandas crescentes requerem a atuação qualificada e compromissada dos líderes na gestão hospitalar. Nesta perspectiva, objetiva-se refletir sobre as práticas de gestão passíveis de serem aplicadas às instituições hospitalares visando o alcance de melhores resultados assistenciais e financeiros. Dentre elas, a gestão baseada em processos propõe uma abordagem para melhoria contínua dos processos a fim de alcançar os resultados desejados; o método *Lean Six Sigma* permite identificar e eliminar desperdícios nos processos produtivos; o modelo de melhoria contínua alia o conhecimento prático ao conhecimento do funcionamento do sistema a ser melhorado, por meio de observações e mudanças que permitam a mensuração de seus resultados; e a gestão de custos e a Saúde baseada em valor preveem que o cuidado seja mapeado, do início ao fim do processo, para avaliar o que, de fato, agrega valor aos pacientes. As contribuições da implementação dessas práticas são reconhecidas mundialmente; utilizando-as, os processos podem ser incrementados, aumentando a eficiência, reduzindo os desperdícios, agregando valor ao negócio, aumentando a sua receita e resultando em economias que podem ser repassadas ao consumidor, pela melhoria da qualidade.

## RESUMEN

Los recursos limitados destinados al área de Salud y las crecientes demandas exigen la actuación calificada y comprometida de líderes en la gestión hospitalaria. En esta perspectiva, el objetivo es reflexionar sobre las prácticas de gestión que se pueden aplicar a las instituciones hospitalarias para lograr mejores resultados asistenciales y económicos. Entre ellos, la gestión basada en procesos propone un enfoque de mejora continua de procesos para lograr los resultados deseados; el método *Lean Six Sigma* permite identificar y eliminar los desperdicios en los procesos productivos; el modelo de mejoría continua combina el conocimiento práctico al conocimiento del funcionamiento del sistema que se reta mejorar, a través de observaciones y cambios que permitan la medición de sus resultados; y la gestión de costes y de salud atención médica basada en el valor establece que la atención sea mapeada, desde el principio hasta el final del proceso, para evaluar lo que efectivamente es lo que agrega valor a los pacientes. Las contribuciones de implementación de esas prácticas son reconocidas a nivel mundial; con ellas se pueden incrementar los procesos, aumentando la eficiencia, reduciendo los desperdicios, agregando valor al negocio, aumentando sus ingresos y generando ahorros que pueden transferirse al consumidor, al mejorar la calidad.

## INTRODUCTION

In the Health area, when discussing the dimensions involving care, management, and administration processes, in view of the growing demands of the population and limited and insufficient resources, it becomes essential, due to their complexity and specificity, to address issues associated with them that interfere, directly or indirectly, in the production of health services.

Considering the Brazilian politics conjuncture, in which the resources destined to Health are increasingly scarcer, the csommitment of leaders, be they from public, private or philanthropic institutions, at different levels of action, with the model of organizational management, cost management and its impacts on the feasibility of different work processes, is crucial for the provision of safe, efficient, effective, and economically sustainable health services.

The COVID-19 pandemic has created challenges for health systems, as it has increased emergency demand for health services, regardless of their legal nature. The hospital network structure, already eroded in some aspects in public and philanthropic institutions, required an increasing number of beds, notably those for intensive care and, consequently, of higher costs, requiring planning and reflection on how to enable the provision of assistance required. The reorganization prioritized care for COVID-19 victims, to the detriment of elective surgeries and outpatient care, which in the medium and long term may imply a pent-up demand for procedures that can lead to the worsening of the population’s health conditions, as well as of the organizations’ finances balance. To overcome this problem, revisions to the models of management, access, processes, and financing policies will be necessary^(1)^.

In Brazil, the financing of primary care has evolved over the years to transfers *per capita*, but hospital care still maintains the fee-for-service model, whose values are standardized by the Unified Table of Procedures of the Brazilian Public Health System (*SUS*), with lagged values. Currently, University Hospitals (*HU*s) represent the largest share of *SUS* care, and it is estimated that they consume 70% of public expenses. However, with the funding crisis worsening, including that for Universities, part of the amounts spent is paid with costing fund. Thus, managing costs becomes of paramount importance, with regard to internal reinvestment and future sustainability policies^(2)^.

Regarding the search for operational excellence, many private institutions, which are classified as philanthropic, because they have some connection with *SUS* activities, present levels of excellence of international standards, but access is still for a small part of the population. Over time, Brazil has maintained funding focused on the Supplementary Health System (*SSS*), to which only 23% of the population has access, which becomes an unequal process. There has been a growing federal incentive for the creation of popular health plans, but with lower-cost disease coverage, and the treatment of severe diseases ends up being expensive for the SUS, which, in turn, is not reimbursed^(1)^.

In this complex context, aiming at the proper functioning of the *SUS*, it appears that Brazil still needs to improve in what regards sufficient funding and adequate management of the resources obtained. Costing should deserve central attention from the Brazilian government, but the fundamental challenges to be faced, for greater efficiency in health care, are concentrated on four aspects: financing, management, access, and workers’ qualification. Specifically in relation to improving management and reducing costs, the introduction of technologies and intelligence in Health is essential^(3)^.

It is reiterated that the demands of health services have been increasing and one of the challenges for hospital management is to balance best care practices with financial sustainability. Therefore, it becomes important to incorporate new working methods and tools, evaluating and improving processes that work properly and checking for the possibilities of improvement, with the introduction of new Health technologies and management tools^(4)^.

Faced with this challenging and complex scenario, marked by an important socio-political-sanitary crisis, the present theoretical-reflective study was proposed, based on national and international literature, guided by the following question: what are the main possibilities to implement evidence-based practices for hospital management aimed at improving work processes? In this perspective, the objective of this study is to reflect on the management practices that can be applied to hospital facilities to achieve better care and financial results. Based on the above, process-based management, the method *Lean Six Sigma*, continuous improvement, and value-based healthcare and cost management will be addressed.

## PROCESS-BASED MANAGEMENT

Process-based management (Business Process Management – BPM) is an approach aimed to improving business processes so that desired results can be achieved through continuous improvement^(5)^. It seeks organizational effectiveness, promoting strategic alignment with the tactical and operational levels, based on management professionalization^(6)^.

BPM brings strategic levels closer to the operational base and reveals how the areas and their systemic interactions, which result in a process design, are really working, and how this cohesion adds value to the business. BPM value is on the standardization of activities and practices, since it establishes how the process should work and those responsible for executing the process are indicated, defining shared responsibilities for the expected final result, with no fragmentation, contributing to efficiency and productivity of the services produced.

BPM increases the quality of care, as processes monitoring takes place through systemic indicators and quality is managed both by the local manager and by the highest institutional hierarchical level, supported by the strategic map (*Balanced Scorecard* – BSC), culminating in top management indicators: learning and growth, internal processes, customers, market and sustainability^(6)^. It focuses externally on the changes taking place in business sectors compatible with their core activities that are measured and compared, through benchmarking, to evaluate process(es) performance, based on indicators, with a view to operational excellence^(5)^.

BPM management requires continuous process improvement, and uses methods, techniques and software systems to analyze operational processes and the respective activities, enabling organizations to become more efficient and assertive in achieving their goals and objectives^(6)^.

The performance of a process is related to time, cost, and throughput-related quality. Therefore, managing processes allows us to understand the design from which the results come and to reflect on possible transformations, through performance indicators (*Key Performance Indicator* – KPIs)^(7)^.

Process modeling is one of the main focuses of BPM management, in which there is no finalization of the work, but constant revisiting of each process so that it is reviewed and improved, seeking alignment between execution and results. Its implementation seeks to increase business competitiveness to eliminate waste, optimizing the productivity of the workers and of the analyzed process itself. It brings technological improvement, as it increases the execution speed and the potentiality of processes, guiding management based on results metrics.

Therefore, robust management methods and technological support are necessary to generate information that helps clarifying what will be the best direction for the managed business. Hospital management should really be thought of as a business, even in the case of public institutions, since it will need to remain financially sustainable, so as not to compromise its costing fund and to foster the prospect of reinvestment policies.

The logic of professional advancement methods, professional allocation, and workforce profile has to be reconsidered, so that there is even use of expertise that brings benefits to the business. Thus, the implementation of corporate and clinical governance models, meritocracy policies, compliance with the law, cost management by a cost center, alignment of business processes and rules,indicators-based management, comparison between similar institutions, knowledge to use management tools will be among the defining topics for adequate operational performance.

Regarding care dimension, the implementation of a clinical governance process subsidizes the measurement of the performance and repercussion of cost management, allowing the management of care protocols and the analysis of the content and time spent in each stage of treatment, aiming to obtain the best results with the lowest costs. The managed protocols incorporate the available scientific evidence, guide the decision-making process based on the clinical outcome (days of stay, surgery time, use of supplies, among others), direct the allocation of health technologies where they are really necessary, and make practices homogeneous^(8)^.

It should be noted that evaluating performance to increase productivity in the *SUS* is quite complex, since it is organized in unequal realities. However, it is necessary to seek improvements in the scope of processes in public health services, with alternatives already used in other market niches, to guarantee quality and efficient allocation of resources, with financial rationalization, expansion of patients’ access to services, and reduction of waste^(7)^.

## LEAN SIX SIGMA

In Brazil, there is a strict certification that conceptualizes hospitals as institutions of excellence, with the way in which they use available resources to produce treatments and services being a decisive element in determining their efficiency. A model of operational excellence, originated in the automotive industry, which has been used in the Health area, is known as lean manufacturing^(9)^.

Lean manufacturing allows identifying and eliminating waste in production processes, with the main focus being to add quality and deliver to the customer (patient) only what he/she considers as value, with an efficient and waste-free process, providing services that respect and meet their preferences and needs^(9–10)^.

The lean model is an industrial philosophy that has been adapted to the Health area, advocating the review of processes to generate value for the patient, balancing and optimizing the required resources. This is associated with improving the workflow, reducing waste, improving patient and professional satisfaction, improving the quality of care, optimizing the physical structure, impacting on transformational leadership and on the improvement of operational and financial results, which directly affect the costs and lead to productivity increase^(11)^.

It is not only considered an improvement tool, but also a method of cultural transformation concerning organizational functioning. Therefore, it requires a process of structural change from top management to the production chain, and it is not a final destination in itself, but a path of continuous search for improvements and operational excellence. In the provision of health services, it takes place in a way that creates a culture that applies the scientific method to design, execute and continuously improve the work provided, focusing on the value that is measurable for the patient^(12)^.

The main impacts arising from the application of this thought in health are: increased productivity and team efficiency; reduction in patient waiting time for care; standardization of care processes; cost reduction; improved teamwork; reduction in patient hospitalization time; improved quality of the service provided; increased patient satisfaction; increased safety for patients and healthcare professionals; and satisfaction of these professionals^(10)^.

The Six Sigma method is a combination of *Lean* System developed by Toyota and adopted by Motorola in 1980, aiming at aligning the operational chain in such a way that value was added to the processes, with waste elimination^(13)^.

*Six Sigma* originated in the United States of America, in 1986, and is based on data analysis to solve problems in order to eliminate waste and optimize processes^(9)^. The creators of STP began to systematically eliminate waste from the production chain, first attacking visible waste until reaching the non-visible waste. This process is guided by two questions, “Where is the waste?” and “What is the best way to get rid of it?”^(14–15)^.

Waste is any activity that consumes resources and does not add value to the product. It corresponds to any and every resource spent on the execution of a product or service beyond what is strictly necessary^(4,16)^. Waste, or activities that do not add value (NAV), can be exemplified as follows: wasting labor or knowledge; defects in the final product or final products in need of repair; stocks that have to be worked on; excess overproduction; waiting time/people waiting for things to arrive; unnecessary human movement; activities that do not add value to the process, but that shall be carried out^(4)^.

The reduction of NAV activities makes the company more competitive. However, it requires a paradigm shift, with the construction of a new culture where the focus is on the customer and processes are designed to meet what adds value (AV), and problems are perceived as opportunities for improvement. In this perspective, *Lean* proposes participatory management, with responsibility and power sharing, and greater involvement of workers in decision-making^(15)^.

In the *Lean method*, the process is designed so that the provision of health services to the client occurs with no deviations, eliminating NAV activities, increasing productivity and efficiency. It adds maturity to management, as it goes beyond the departmental view and creates a process-oriented culture, which can stimulate people’s commitment and deliver benefits to the business. Therefore, it is necessary to be clear about the intended final result, to assign those responsible for the processes sustainability, and to measure performance based on these^(10)^.

A study highlights that the interactions between the operational and strategy teams are fundamental for improving the work process and encourage listening and professional appreciation, due to the feeling of collectivity. This way, there is good use of talents of the teams’ members who, duly trained to use the methodology, perceive its applicability in other processes in the same workplace, transforming the realities with the reduction of time and financial resources spent, qualifying the work process^(17)^.

The method *Lean Six Sigma* is based on six principles^(17)^:

– Continuous improvement: it is a scientific method applied to daily work, where an explicit and measurable hypothesis is defined on how a process can be improved. This hypothesis is tested until improvements can be demonstrated;
– Value creation: value in Health is understood more broadly, including monetary values and non-monetary costs. The benefits that add value to the patient go beyond the financial construct to include patients’ perceptions of the overall health care experience. In this context, value stream mapping (VSM) is a tool used to distinguish between steps in a process that add value or not. VSM is built together with the team, containing each step of an existing process, to better understand it regarding its current state and what has to be essentially improved;
– Establishing priorities: the management process prioritizes clear communication through indicators of strategic goals that are relevant to the entire organization and that provide an opportunity to strengthen the organization and create value;
– Respect for the people who do the work: the lean management empowers and gives voice to frontline workers, who become protagonists of innovation, and managers assume the role of trusting, directing, supporting and enabling. Management shall design a process in which a safe environment is created so that workers do not fear reporting problems, are safe to innovate, with the focus being to attack obsolete processes and not people;
– Visual: demonstrate, through graphs, data and indicators that are relevant to the results achieved, as well as the established goals and planned actions;
– Flexible regimentation: the essence of *Lean* is looking at a non-standard process and making it standard, improving performance, even in the optimal state.

It should be noted that the method *lean* contributes to BPM management, as it is aimed at reducing process variability, contributing to standardization and increased productivity. At first, relating productivity to health services can generate a direct connotation to workforce; however, it is emphasized that the lean thought focuses on how the process is designed so that the workforce becomes truly productive, eliminating bottlenecks, unnecessary steps and displacements and reworks, which are associated with waste in the process. It aligns the strategic levels for operation management that have to guide the processes for the best results, from the production base, eliminating sectorization and rigid hierarchies, seeking greater fluidity to the mapped process.

## CONTINUOUS IMPROVEMENT

Continuous improvement has driven companies to seek programs to qualify products and services. Quality management, as an approach to improving competitiveness, effectiveness, and flexibility of the organization as a whole, was introduced by Edwards Deming in 1986^(18)^. One of Deming’s quality principles is process-guided management, with the concept of the “supplier-process-customer” chain, integrating different functions and composing an interaction network. To apply the improvement model, it is necessary to combine practical knowledge with knowledge on the functioning of the system to be improved, through observations and changes that allow its results measurement^(19–20)^.

PDSA (*Plan-Do-Study-Act*) cycles structure the development of changes, either as a stand-alone method or as part of broader quality improvement approaches (for example, the first principle of the method *Lean Six Sigma*).

A systematic review showed that PDSA cycles offer a mechanism to support the development and scientific testing of improvements in complex health systems. Its use is considered, in itself, a complex intervention consisting of a series of interdependent steps and key principles that inform its application, which is also affected by the local context. Therefore, to interpret the results of the PDSA cycles, it is necessary to understand how the strategies were implemented^(21)^.

Users of the PDSA method follow a prescribed four-step cyclical learning approach to adapt changes aimed at improvement. In the step Plan, an improvement-oriented change is identified; in the step Do, this change is tested; the step Study examines the success of change through data and indicators, and the step Act identifies adaptations and next steps to inform a new cycle. Compared to more traditional methods of health research, the PDSA cycle presents a pragmatic scientific method for testing changes in complex systems. The four steps reflect the scientific experimental method of formulating a hypothesis, collecting data to test that hypothesis, analyzing and interpreting the results, and making inferences to iterate on the hypothesis^(22)^.

The principles of PDSA cycles promote the use of an iterative, small-scale approach to testing interventions, as this allows for rapid assessment and provides flexibility to adapt change according to the *feedback* to ensure that suitable solutions are developed. It is recommended to start implementing strategies with small-scale tests. This way, users are free to act and learn; minimize risk to patients, to the organization and to resources needed, and provide the opportunity to build evidence to scale up change and engage stakeholders as confidence in interventions increases^(19–22)^.

In line with the scientific experimental method, PDSA cycles promote the prediction of the outcome of a test of change and subsequent measurement over time (quantitative or qualitative) to assess the impact of an intervention on the process or outcomes of interest. Thus, learning is achieved primarily through interventional experiments designed to test change. In respect of work in complex environments with inherent variability, measuring data over time helps to understand natural variation in a system, increase awareness of other factors that influence processes or outcomes, and understand the impact of an intervention^(12,22)^.

PDSA cycles can be well applied when there is a problem to be solved, with well-established goals and deadlines. They take place through organizational learning and diagrams are used to guide the actions that need to be implemented to achieve improvement, and these must be based on scientific evidence. After defining the guidelines, these are tested in practice, studied and adapted to each reality, and then the best implemented changes that will have a favorable impact on the results are diagnosed. They are quite effective when there is an already defined structure of the goals to be achieved and they guide the continuous improvement of the processes.

As with all scientific methods, documentation of each step of the PDSA cycles is important to support scientific quality, learning, and local reflections, and to ensure that knowledge is captured to support organizational memory and the transfer of learning to other environments^(22)^.

It is reiterated that the principles presented value customer focus, continuous improvement, teamwork, and continuous updates. One of the assumptions is that quality would be an assertive way of planning, organizing and understanding that each activity depends on each individual and each level of hierarchy. Therefore, a quality program requires structural and cultural change in the company, in the way people work, and even in how they feel about participation in institutional results^(18)^.

## COST MANAGEMENT AND VALUE-BASED HEALTHCARE

Health funding in Brazil has fluctuated in recent years around 8% of gross domestic product (GDP), close to that of developed countries such as Canada and the United Kingdom, which spend 10.4% and 9.9% of GDP, respectively. However, spending larger fractions of GDP on health system funding does not mean better health conditions for the population^(1)^.

Given the current scenario, the challenge for Health systems lies in transforming the form of relationship and incentives of the entire value chain, which needs innovative responses to customer demands, through technology, globalization, social participation, and financial constraints. The way to face this challenge is to place the client in a central position and, consequently, to reorganize the care delivery chain. This approach is called value-based healthcare, focusing on delivering the best outcomes, defined by the customer’s perspective, and at the lowest cost possible^(11)^.

Discussing value-based health is something quite complex, it is not just about value from the client’s point of view, it is about value resulting from the relationship between quantity and quality of care provided and the associated costs. The challenge lies in how to direct a work process that guarantees added value, considered from beginning to end, and that results in better care and financial results, at the lowest cost possible, having waste reduction as the main objective, as this adds value and saves money. The reduction of waste, including that in work processes, can increase quality in a way that contributes to increased financial income and cost reduction^(23)^.

The concept of value-based health comprises care mapped from end to end, from beginning to end of the process, aiming to evaluate what adds value to clients and how this process can be improved from its conception^(11)^.

The modalities of remuneration, based on value (benefits measured by clients in their relationship with health services), combined with the optimization of the economic performance of their production, repackage the cost-benefit and cost- effectiveness analyses of interventions. These can introduce economic performance metrics at the individual care level and will be more successful in implementation if combined with resource allocations calculated based on the volume and overall quality of services. Pressures to reduce costs and increase services safety and quality will be opposed to the need to guarantee minimum levels of resources to service providers, under penalty of causing their financial insolvency by the escalation of requirements of all kinds^(1)^.

A change that adds value is one that improves care using fewer resources, or the same resources, by reducing waste and applying new technologies. It should be noted that better quality alone does not add value, given that value is based on quality in relation to the resources used. Moreover, for this approach to be successful, it is necessary to adapt experiences that demonstrate effectiveness to the particular context of each organization, favoring personnel training for people to work in different ways, which support productivity^(22)^.

The Cost-Expenses-Savings (CES) has been a model to estimate whether an improvement adds value. As it includes Return on Investment (ROI), defined as any and every resource (financial, human, or a purpose) that returns in a way that adds value to something, it provides a way to assess the costs of the problem, the costs with the solution and savings, the resulting loss or additional financial resources. CES serves to monitor the financial progress of the implemented change, which can be a persuasive subsidy in favor of saving resources. Through it, financial estimates are made in three aspects: cost of the problem related to quality (annual cost estimate); expenses to reduce the problem by 50% (estimation of the costs of evaluating and monitoring the problem); and savings/losses in one year and in subsequent years (progressive demonstration of how much the organization can save in the years following the implementation of improvement strategies)^(23)^.

In hospital practice, value-based health has been applied in association with clinical governance models, to improve care results and reduce waste, through monitoring of clinical outcomes and rational allocation of required resources. It is shown to be an efficient form of reorganization, as it directs what is important to add value to the client and the economic-financial return for the institution, showing possibilities of how to serve the client at the lowest cost possible, without compromising quality. In this perspective, the use of artificial intelligence to measure the complexity and criticality of patient care is currently observed, allowing the performance of predictions and projections on care results.

## CONCLUSION

The reflections on the management practices addressed in the present study aimed to highlight their potential to contribute to the increase of care and management results, which can have a favorable impact on the economic-financial aspects of public, private, or philanthropic hospital organizations, inserted in a scenario of rising costs and expenses, linked to the challenges of financing health systems.

However, it is reiterated that the achievement of institutional goals, although important, is not the end in itself, but a means to seek continuous improvement. Therefore, process-based management proposes an approach for continuous process improvement to achieve desired results; the method *Lean Six Sigma* allows identifying and eliminating waste in production processes; the continuous improvement model combines practical knowledge with the knowledge of how the system to be improved works; and cost management and value-based healthcare provides for care mapping, from beginning to end, to assess what actually adds value to the clients.

It should be noted that the contributions of implementing these practices are recognized worldwide and, by implementing them, high hospital management improves processes efficiency, reducing waste, adding value to the business, increasing its revenue, and resulting in savings that can be passed on to the consumer, by continuous quality improvement.

It is therefore necessary to equip managers for the proper use of evidence-based knowledge, specific to management, focusing on measurable and financially sustainable results, with researches that support the reorganization of care and management processes being essential.

## ASSOCIATE EDITOR

Paulino Artur Ferreira de Sousa
